# Bacterial Resistance to β-Lactam Antibiotics in Municipal Wastewater: Insights from a Full-Scale Treatment Plant in Poland

**DOI:** 10.3390/microorganisms10122323

**Published:** 2022-11-24

**Authors:** Natalia Jendrzejewska, Ewa Karwowska

**Affiliations:** Department of Biology, Faculty of Building Services, Hydro and Environmental Engineering, Warsaw University of Technology, Nowowiejska 20, 00-653 Warsaw, Poland

**Keywords:** antibiotic resistance, multi-resistance, resistance genes, β-lactamases, wastewater treatment, microbial community

## Abstract

This study investigated enzymatic and genetic determinants of bacterial resistance to β-lactam antibiotics in the biocenosis involved in the process of biological treatment of wastewater by activated sludge. The frequency of bacteria resistant to selected antibiotics and the activity of enzymes responsible for resistance to β-lactam antibiotics were estimated. The phenomenon of selection and spread of a number of genes determining antibiotic resistance was traced using PCR and gene sequencing. An increase in the percentage of bacteria showing resistance to β-lactam antibiotics in the microflora of wastewater during the treatment process was found. The highest number of resistant microorganisms, including multi-resistant strains, was recorded in the aeration chamber. Significant amounts of these bacteria were also present in treated wastewater, where the percentage of penicillin-resistant bacteria exceeded 50%, while those resistant to the new generation β-lactam antibiotics meropenem and imipenem were found at 8.8% and 6.4%, respectively. Antibiotic resistance was repeatedly accompanied by the activity of enzymes such as carbapenemases, metallo-β-lactamases, cephalosporinases and β-lactamases with an extended substrate spectrum. The activity of carbapenemases was shown in up to 97% of the multi-resistant bacteria. Studies using molecular biology techniques showed a high frequency of genes determining resistance to β-lactam antibiotics, especially the *bla*TEM1 gene. The analysis of the nucleotide sequences of *bla*TEM1 gene variants present in bacteria at different stages of wastewater treatment showed 50–100% mutual similarity of.

## 1. Introduction

Bacterial resistance to antibiotics is one of the most serious challenges of the 21st century [1,2,3]. The growing phenomenon of drug resistance in potentially pathogenic microorganisms contributes significantly to the decreased effectiveness of antimicrobial therapies [4,5]. There is a successive increase in the number of drug-resistant strains and a decrease in the number of antibiotics with sufficiently effective action [6]. It is estimated that by 2050, antibiotic resistance in microorganisms could contribute to the deaths of up to 10 million people worldwide [7].

The widespread use of antibiotics and their consequent presence in wastewater, not only from the pharmaceutical industry but also increasingly from agriculture and individual households, is leading to the development of microbial populations capable of developing defense mechanisms that allow them to survive in environments containing these substances [8]. The pathogenic and potentially pathogenic bacteria released into the aquatic environment with wastewater often possess antibiotic-resistance genes located within mobile genetic elements, allowing them to spread more efficiently among bacteria present in water bodies [9]. These processes are particularly enhanced at sites where significantly higher concentrations of pharmaceuticals are present, e.g., in surface water near wastewater discharges or agricultural waste disposal sites [9,10], although a transfer of drug-resistance conditioning plasmids has also been shown to occur in the absence of pharmaceuticals in wastewater [11]. The range of impact of a point source of environmental pollution with microorganisms carrying resistance genes can reach many kilometers [12].

The so-called horizontal gene transfer is one of the mechanisms that, in addition to spontaneous or induced mutations within the bacterial genome, enables not only the interspecies spread of antibiotic resistance [13], but also the accumulation of genes responsible for them, leading to the formation of multi-resistant strains. Mobile genetic elements such as plasmids, transposons or integrons that have embedded genes, or even entire antibiotic-resistance gene complexes, are transferred from cell to cell through the processes of conjugation, transduction or transformation [2,14]. 

Due to the varying sensitivity of individual microbial species to specific antibiotics, these substances can have a key impact on the structure of bacterial communities in the environment [15,16,17]. The change in conditions induced by the presence of an antibiotic usually results in a decrease in the microbial biodiversity [18,19], although sometimes an intense proliferation of strains, previously present in low numbers while possessing the appropriate mechanisms for drug resistance, can be observed [20]. At very high concentrations of antibiotics, the ecosystem can become populated with resistant microorganisms that would not otherwise be present [21]. Such changes in the bacterial microflora can be long-term and persist long after the contamination has disappeared from the environment [22]. 

Technological systems used for biological wastewater treatment are the habitats of a biocenosis specialized in the degradation of specific types of pollutants. Changes within them, leading to the replacement of some bacterial populations by others, are very important in this context. The natural structure of bacterial communities is reconstructed with difficulty after their exposure to antibiotics, and generally does not return to its initial state. As a result of the pressure caused by the presence of antibiotics, some functions encoded in the bacterial genome can be permanently lost, especially those responsible for an adaptation or those specific to individual microbial cells. This can lead to the replacement of some bacterial populations by others. This is particularly relevant for sites such as wastewater-treatment plants, especially when it involves changes within a biocenosis adapted to the degradation of a particular type of pollutant [23]. It can strongly affect the efficiency of treatment processes. In turn, the selection of drug-resistant microbial strains represents an additional secondary contaminant released into the environment with treated wastewater. What differentiates such “biological contamination” from chemical pollution is the ability to autoreplicate (and thus persist), and the ability to move and spread [24]. Microbial communities that form activated sludge or a biofilm are convenient sites for the spread of drug resistance [25,26,27,28]. Conditions at wastewater-treatment plants (including a constant supply of pharmaceuticals and a high density of microorganisms) promote gene transfer between bacteria present in wastewater and the persistence of antibiotic resistance [29,30]. The wastewater-treatment technologies that are currently in use allow the effective removal of traditional pollutants, but they are not designed to remove antibiotic residues [31], much less eliminate drug-resistant bacteria and antibiotic-resistance genes [3]. 

The aim of the present study was to trace the enzymatic and genetic determinants of bacterial resistance to β-lactam antibiotics in the bacterial biocenosis present in wastewater from a municipal wastewater-treatment plant. A quantitative study of bacteria resistant to selected antibiotics, as well as a study of the activity of enzymes responsible for resistance to β-lactam antibiotics, was carried out. The phenomenon of selection and spread of certain genes determining antibiotic resistance was traced using PCR analysis and gene sequencing.

## 2. Materials and Methods

**Figure 1:**
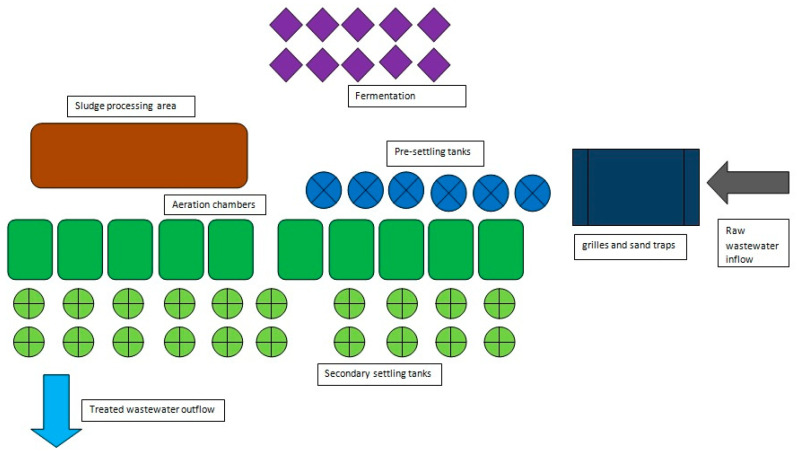
The schematic of the wastewater-treatment plant used as a source of the wastewater samples in the study.

The wastewater sampling site used in this study is one of the largest wastewater-treatment plants in Poland, with a capacity of about 435,300 m^3^ of wastewater per day. It is a mechanical–biological treatment plant with enhanced removal of nutrients. The biological treatment stage is carried out within 10 process lines, each of which includes a biological reactor equipped with aeration chambers, two secondary settling tanks and an activated-sludge recirculation system. After treatment, the wastewater is discharged into the Vistula River. The schematic of the wastewater-treatment plant is presented in Figure 1.

The experimental work was carried out for three years. During this time, six series of measurements were made. Samples for the study were taken from wastewater entering the treatment plant (4 different inflows), from the aeration chambers and from the wastewater stream after the treatment process. The samples were transported to the laboratory under refrigerated conditions and subjected to microbiological analysis.

The quantitative studies of bacteria were carried out on nutrient agar medium (to determine the total number of bacteria), and on selective media with the addition of β-lactam antibiotics such as penicillin, ampicillin, imipenem and meropenem (Biocorp Poland Ltd., Lab Empire s.c., Rzeszów, Poland). To prepare the selective media, a suspension of the antibiotic in distilled water was added to nutrient agar media that had been cooled to about 50 °C. The antibiotic was added in an amount to obtain the appropriate concentration: for penicillin and ampicillin–40 mg/L, for imipenem and meropenem–20 mg/L, respectively. The concentrations used were slightly higher than the MIC values reported by EUCAST (2021); this was in order to ensure that only definitely antibiotic-resistant strains would be cultured. Therefore, the selection of antibiotic concentrations was based on data from the literature [32,33]. Surface culture of samples was performed on Petri dishes with appropriate media. The cultures were incubated at 37 °C for 48 h. Bacterial counts were reported as the number of colony-forming units (CFU) in 1 cm^3^ of wastewater. By comparing bacterial counts on individual selective media with added antibiotics with the total number of bacteria in the wastewater, the percentage of antibiotic-resistant bacteria was determined before, during, and after the wastewater treatment.

In order to obtain pure isolates of β-lactam antibiotic-resistant bacteria, the predominant bacterial strains were selected from among those obtained on media with the addition of individual antibiotics. Antibiotic-resistant bacteria were isolated in individual series from antibiotic-specific media plates that had been used during the quantitative testing. Despite their abundance, the antibiotic-resistant bacteria obtained were characterized by a relatively low diversity as suggested by the low variation in the colonies’ morphology. Strains differing in colony morphology were subjected to purification by the passage method, and then identified using standard biochemical tests: API E and API NE (bioMerieux Polska Ltd, Warsaw, Poland. Using the passage technique on media with different antibiotics, it was determined which of the isolates showed characteristics of multi-resistance. 

The enzymes involved in the antibiotic resistance—carbapenemase, metallo-β-lactamase, cephalosporinase and β-lactamase of extended substrate spectrum (ESBL)—were assessed using the double-disc method (ROSCO). The presence of the enzymes was evaluated basing on the zone diameters around the discs with meropenem, meropenem + phenylboronic acid (carbapenamase inhibitor), meropenem + dipicolinic acid (metallo-β-lactamase inhibitor), and meropenem + cloxacilin (AmpC inhibitor). Average values of five determinations were calculated. The results were interpreted according to the test manufacturer’s instructions.

In addition, in order to determine the presence of carbapenemases in the tested bacterial isolates, the Blue-Carba test was used, which allows the detection of acquired carbapenemases in Gram-negative bacteria, including those of the *Enterobacteriaceae* family and bacteria of the *Pseudomonas* genus. A significant part of the tested isolates belonged to the *Enterobacteriaceae* family, while some isolates were identified as belonging to the genus *Pseudomonas*. The principle of the test is to observe the hydrolysis of imipenem caused by carbapenemases in a suspension of bacterial cells. To perform the assay, 0.01 cm^3^ of a 24-h bacterial culture was introduced into 0.2 cm^3^ of a reaction mixture containing imipenem (3 mg/cm^3^) in 0.04% bromothymol blue solution, pH 6.0, with the addition of 0.1 mM zinc sulfate. The control sample prepared in parallel was a bromothymol blue solution at pH 7.0 with 0.1 mM zinc sulfate, inoculated with a similar number of bacteria, without the addition of imipenem. The inoculated samples were then incubated for 2 h at 37 °C. The test result was read according to the test interpretation rule given by Pires et al. [34].

The isolation of bacterial DNA was carried out using EXTRACTME DNA BACTERIA KIT BLIRT S.A.Gdańsk, Poland) reagent kit according to manufacturer’s instruction. The isolated DNA was stored at −20 °C.

The amplification of bacterial DNA using PCR reactions was carried out using dedicated kits and reagents: REDTaq^®^ ReadyMix™ PCR Reaction Mix (Sigma-Aldrich, St. Louis, MO, USA) and GoTaq^®^ Green Master Mix (Promega, Madison, WI, USA). DNA size markers from GenoPlast Biochemicals (Rokocin, Poland) and PCR and qPCR reaction primers from Syngen Biotech Ltd (Wrocław, Poland) were used (Table 1). The reaction included initial denaturation (3 min at 95 °C) and 35 cycles consisting of denaturation at 95° for 30 s, primer attachment at 52–66 °C (depending on the primer) for 30–60 s, polymerase attachment to the primer duplex and chain synthesis at 72 °C for 1 min, and final extension at 72 °C for 10 min. 

The resulting amplification products and DNA mass standards were applied to an agarose gel immersed in TAE buffer (pH 8.0) and electrophoresed at 100–110 V for about 40–60 min. The concentration of agarose was chosen according to the size of the expected DNA product. The gel was then washed in ethidium bromide solution. Observation under UV light was carried out and photographic documentation was made. 

In order to examine the actual copy number of the *bla*TEM1 gene, the qPCR method was applied, using the KAPA SYBR^®^ FAST qPCR Master Mix (2X) Kit reagents (Sigma-Aldrich). The reaction included initial denaturation (3 min at 95 °C) and 40 cycles consisting of denaturation at 95° for 15 s, primer attachment at 60 °C for 20/60 s for TEM/16S, respectively, followed by 30 s at 72 °C. The reaction products were analyzed using Applied Biosystems StepOne Software Version 2.1. The copy number of the *bla*TEM1 gene was related to the determination of the total number of bacteria (expressed as the number of copies obtained with the 16S primer).

The sequencing of the obtained PCR products was accomplished by Genomed (Warsaw, Poland), by real-time pyrosequencing, based on Roche’s GS Junior system. 

The analysis of sequencing results was carried out using BLASTN software, with the help of databases provided online by services: ARDB-Antibiotic Resistance Genes Database, BLAST- Basic Local Alignment Search Tool, and Genome- BLAST Search.

## 3. Results

The quantitative studies of the microflora from samples taken at various stages of the wastewater treatment showed that bacteria characterized by resistance to ampicillin, penicillin, as well as new-generation antibiotics such as imipenem and meropenem, were present in large numbers in both raw wastewater delivered to the treatment plant and in treated wastewater, and in wastewater discharged to the receiver. The abundance of antibiotic-resistant bacteria in the samples tested was 10^4^–10^5^ CFU/cm^3^ in the raw wastewater and in the wastewater collected from the aeration chamber, and about 10^3^ CFU/cm^3^ in the treated wastewater. The number of the bacteria capable of growing in the presence of antibiotics remained similar throughout the study. The standard deviation accounted for 7–19% of the number of bacteria determined in raw wastewater and wastewater in the aeration chamber, and 1.8–6.5% for treated wastewater (for different antibiotics used). The lowest counts of the antibiotic-resistant bacteria were found in the second year of the study.

**Figure 2:**
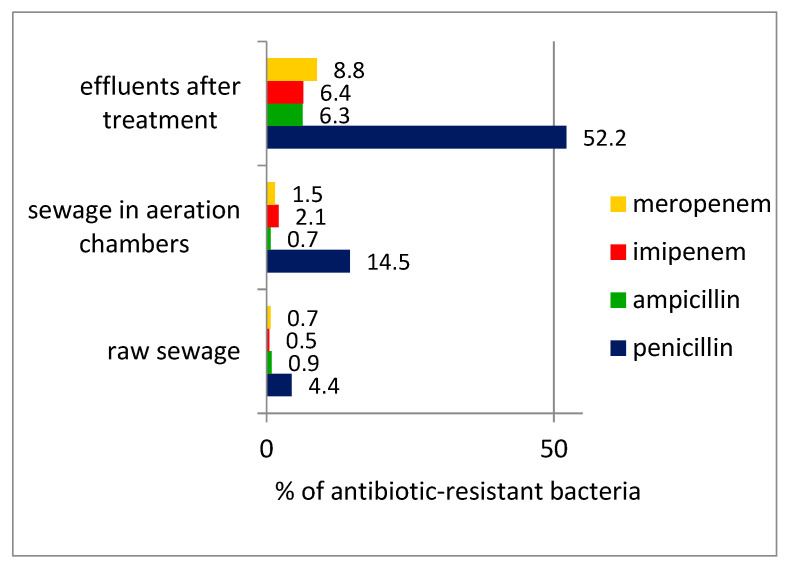
The percentage of antibiotic-resistant bacteria of the total bacterial number.

During the wastewater-treatment process, there was a significant increase in the percentage of β-lactam antibiotic resistant bacteria in the treated wastewater compared with the raw wastewater, confirming the possibility of spreading drug-resistance traits during the wastewater-treatment process (Figure 2).

Among the predominant bacterial strains isolated from wastewater samples before, during, and after the treatment process, a total of 128 pure isolates of bacteria resistant to the β-lactam antibiotics tested were obtained, with more than half of them (70 isolates) being multi-resistant strains. The number of isolates obtained at different stages of the wastewater treatment was: 59 strains from raw sewage, 31 strains from aeration chambers and 38 strains from the effluent. The corresponding numbers for year 1-3 were 17, 86, and 25 strains, respectively. Although dominant bacterial strains were isolated each time, it should be noted that the second year of the study showed an increased diversity of antibiotic-resistant bacteria with relatively lower numbers. 

The strains of the predominating antibiotic-resistant bacteria belonged to species: *Escherichia coli*, *Pseudomonas putida*, *Pseudomonas luteola*, *Burkholderia cepacia*, *Aeromonas hydrophila*, *Acinetobacter baumanii*, and *Stenotrophomonas maltophilia*. Some strains of the *Salmonella* and *Serratia* genera were also isolated. A comparison of the results obtained collectively for the entire period analyzed (3 years) showed that the average number of antibiotics that were ineffective against multi-resistant strains isolated from aeration chamber and treated wastewater was comparable to the value obtained for raw wastewater, and in some cases even higher, which may suggest an increase in the scale of microbial resistance in the course of the wastewater-treatment process. 

**Figure 3:**
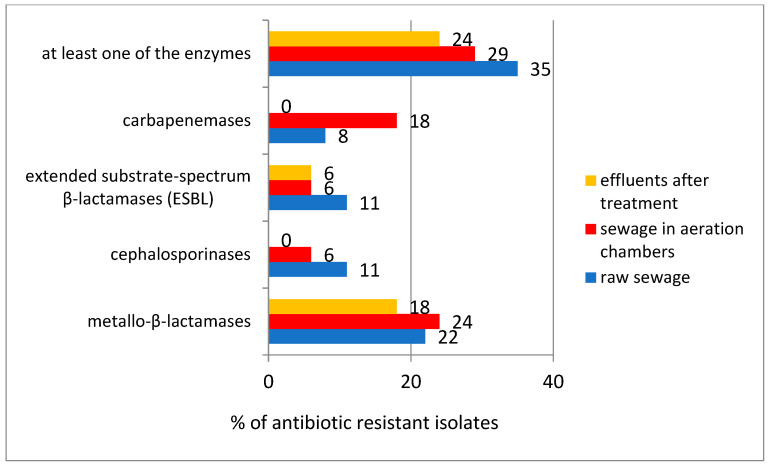
The frequency of occurrence of antibiotic-resistance enzymes in bacteria isolated from raw wastewater, wastewater in aeration chambers and wastewater after the treatment.

In the double-disc enzymatic assay, the presence of metallo-β-lactamases was found in 20% of the tested isolates, while carbapenemases, cephalosporinases and extended substrate spectrum β-lactamases (ESBLs) were detected in about 10% of the tested strains. The percentage of bacteria showing the activity of at least one enzyme from the tested group was on average: in raw wastewater—35%; in wastewater taken from the aeration chamber—29%; in treated wastewater—24%; there was no significant increase in the percentage of strains showing this type of enzymatic activity during the wastewater-treatment process (Figure 3).

Significantly higher results were obtained by the Blue-Carba test. It was found that bacteria alone showing carbapenemase activity accounted for about 50% of the strains present in raw wastewater, and 35% and 29% in aeration chamber wastewater and treated wastewater, respectively. It should be noted that in the case of strains previously found to be multi-resistant, a positive Blue-Carba test result indicating the carbapenemase activity was recorded in up to 97% of isolates.

**Figure 4:**
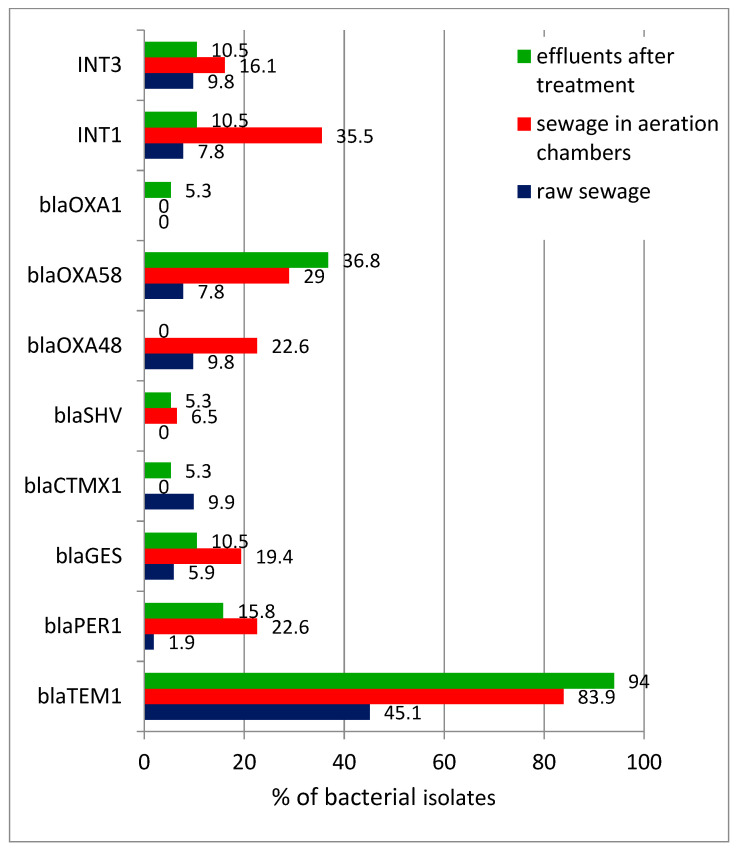
The percentage of antibiotic-resistance genes in bacteria isolated from the wastewater before, during, and after biological wastewater treatment.

The analysis of the PCR reaction products showed the presence of all ten tested genes, (i.e., *bla*TEM1, *bla*GES, *bla*PER1, *bla*OXA58, *bla*OXA48, *bla*OXA1, *bla*CTXM1, *bla*SHV, *Int*1, and *Int*3) in the isolated antibiotic-resistant bacteria (Figure 4). The *bla*TEM1 gene was detected with the highest frequency. It was found that the percentage of isolates possessing drug-resistance genes was already increasing significantly during wastewater treatment (samples collected from the aeration chambers) and, as a result, in treated wastewater it exceeded 90% for *bla*TEM1 gene. A number of strains gave a positive result after using the primers for several resistance genes; this also applied to bacteria isolated from treated wastewater. It should be noted that strains from raw wastewater usually gave a positive result when using one (less often, two) primer for antibiotic-resistance genes; while for the strains from the aeration chamber and then treated wastewater, the number of genes increased to 3–4 (this was true for more than 26% of isolates). Therefore, the study found two effects in parallel— an increase in the percentage of strains containing resistance genes and an increase in the number of genes carried by each strain. The co-occurrence of *bla*TEM1 and the gene encoding integron class 1 (*Int*1) was also found. 

Studies conducted by qPCR confirmed the persistence of a significant number of copies of the *bla*TEM1 gene in the wastewater-treatment process (at 10^5^–10^6^/cm^3^) and the percentage of bacteria equipped with this gene in relation to the total number of bacteria in the wastewater at around 1%. 

**Figure 5:**
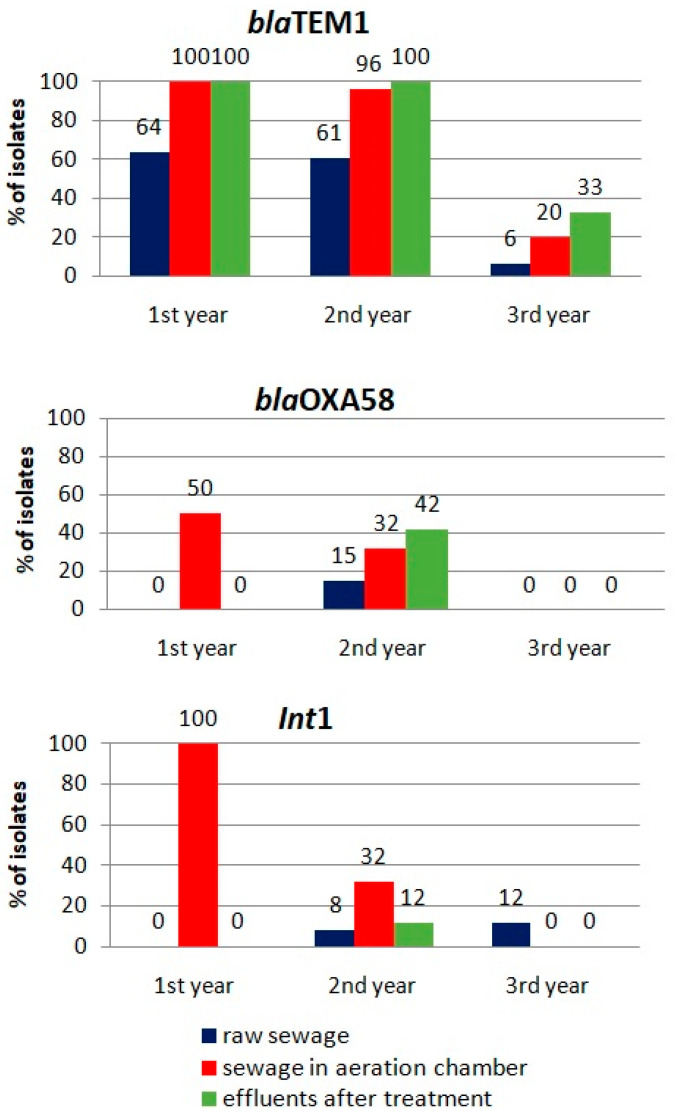
The occurrence of selected genes during the study, at different stages of the wastewater treatment.

For the genes most abundant in the isolated antibiotic-resistant bacterial strains (*bla*TEM1, *bla*OXA58 and *Int*1), an analysis of their occurrence at different stages of the wastewater-treatment process was carried out during the subsequent years of the study (Figure 5). An increase in the incidence of the *bla*TEM1 gene during the wastewater-treatment process was noted based on data acquired in all years. A similar effect was observed for the *bla*OXA58 gene in the second year of the study.

A significant decrease in the frequency of the detection of all analyzed genes occurred in the last year of the study, which was probably a consequence of their less frequent appearance in the raw wastewater flowing into the treatment plant. This confirms the significance of the impact of external sources of antibiotic-resistance factors on the scale of the drug-resistance hazard in technological systems.

**Figure 6:**
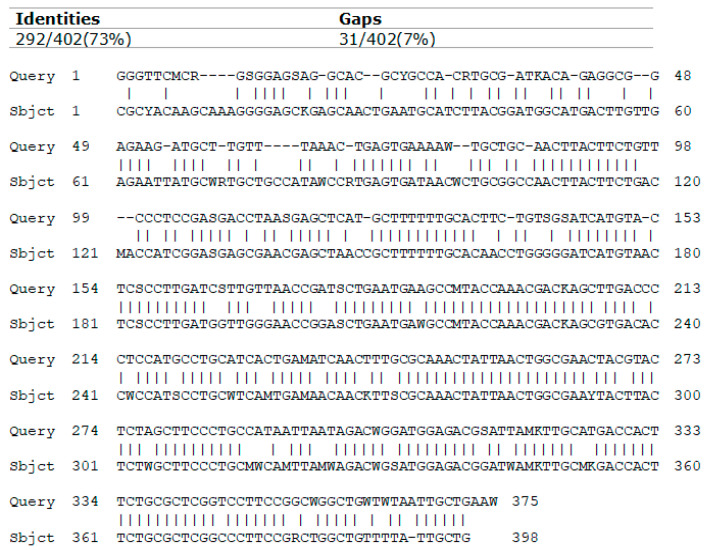
The comparison of *bla*TEM sequences for a sample bacterial strain isolated from raw wastewater (Subject sample) and a strain obtained from wastewater in the aeration chamber (Query sample).

A cross-analysis of the nucleotide sequences of specific versions of the *bla*TEM1 gene in bacteria isolated from raw wastewater, wastewater in aeration chambers and treated wastewater showed that they had a mutual similarity of 50–100%, suggesting the persistence of specific versions of the drug-resistance determinant gene throughout the wastewater-treatment process. A sequence similarity of 100% was recorded for 30% of multi-resistant strains isolated from the aeration chamber. A 68–73% sequence concordance was observed between 40% of multi-resistant strains from the aeration chamber and 13% of strains in raw wastewater (Figure 6). A 50% of similarity was observed between 20% of multi-resistant strains from the aeration chamber and 17% of multi-resistant isolates from the treated wastewater.

## 4. Discussion

A side effect of the increase in the production and widespread use of various pharmaceuticals is the significant development of the phenomenon of drug-resistant microorganisms. The successive increase in the number of drug-resistant bacteria is a problem that scientists around the world are struggling with.

Studies conducted in European wastewater-treatment plants [35,36] confirm the ineffectiveness of conventional treatment methods in reducing the presence of genes that determine microbial antibiotic resistance in wastewater. The availability of nutrients and the significant diversity of microorganisms in activated sludge, crucial for ensuring effective wastewater treatment, simultaneously provide the perfect environment for the maintenance and the spread of antibiotic resistance [37]. Available data suggest that wastewater-treatment plants, which receive a significant portion of antibiotic residues and their metabolites, should be considered as a potential source of secondary environmental contamination with drug-resistant bacteria. Hence, it is important to trace the determinants of the antibiotic-resistance phenomenon in the biocenosis of microorganisms in the wastewater-treatment system, which was the subject of this study. 

In this research, a comprehensive analysis of the phenomenon of β-lactam antibiotic resistance in bacteria present in wastewater during its biological treatment was carried out. Both quantitative studies of drug-resistant microflora and the analysis of the occurrence of important genes determining this resistance were performed. Molecular studies were complemented by the analysis of the activity of enzymes that play a key role in the effective functioning of microorganisms in the presence of antibiotics.

The quantitative studies confirmed the numerous occurrences of β-lactam antibiotic-resistant bacteria in wastewater, and the fact that they accounted for a significant percentage of the total number of bacteria present in wastewater, including wastewater after the treatment process. A similar fact was previously reported by Huang et al. [38], who detected about 10^4^–10^5^ CFU/cm^3^ of penicillin-resistant bacteria in the effluent of a wastewater-treatment plant in China. This seems particularly worrisome that β-lactams are among the most widely used chemical compounds with antibacterial properties. 

For almost all antibiotics, an increase in antibiotic-resistant bacteria was observed in the aeration chambers, that is, while the wastewater-treatment process was still in progress; this is in line with the current literature which reports that these are places where drug resistance can spread [28,29,30]. This is probably because the presence of microorganisms in the form of flocs or biofilm promotes the transfer of genes that determine drug resistance [39].

In the present study, we focused on the resistance of the bacteria that make up the wastewater microflora to β-lactam antibiotics. The study included both traditionally-used β-lactam antibiotics such as penicillin and ampicillin, and antibiotics classified as new generation drugs, namely meropenem and imipenem. It is the genes encoding β-lactamases (including *bla*TEM1) that fall into the highest risk category [40].

The present study analyzed the occurrence of ten genes of this type: *bla*TEM1, *bla*GES, *bla*PER1, blaOXA58, *bla*OXA48, *bla*OXA1, *bla*CTXM1, *bla*SHV, *Int*1, and *Int* 3. 

The *bla*TEM genes, of which more than 100 types have now been identified, represent *bla*TEM1 and *bla*TEM2 variants. They are typically located within transposons associated with drug-resistance traits [41]. The products of these genes are among the extended-spectrum β-lactamases (ESBLs), which are most commonly acquired by hospital isolates of the *Enterobacteriaceae* family worldwide [42,43]. They are responsible for a high percentage of ampicillin-resistance in *E. coli* and *Klebsiella pneumoniae* bacteria [44]. 

The *bla*GES gene encodes an extended-spectrum β-lactamase and is detected in *Klebsiella pneumoniae*, other *Enterobacteriaceae,* and *Pseudomonas aeruginosa* in various regions of the world (including France, Greece, South Africa and Japan). These genes are located mostly on class 1 integrons [45]. 

The *bla*PER gene was found in 8 variants; mainly in Gram-negative bacteria of the *Acinetobacter baumannii, Pseudomonas aeruginosa,* and *Alcaligenes faecalis* species, and in clinical strains of the *Enterobacteriaceae* family. The product of the *bla*PER1 gene was first discovered in the *Pseudomonas aeruginosa* strain RNL-1; it hydrolyzes some oxyimino-cephalosporins, while its activity is inhibited in the presence of tazobactam and clavulanic acid [46].

The spectrum of action of the *bla*SHV1 gene product is similar to that of *bla*TEM1, but it achieves a higher activity against ampicillin. It has been identified in several species of the *Enterobacteriaceae* family [47].

The *bla*CTXM1 genes, first detected in *E. coli*, condition resistance to cefotaxime and ceftazidime. They are carried by mobile genetic elements such as transposons and plasmids [48].

The *bla*OXA genes, which encode oxacillinases, are mainly responsible for conferring resistance to cephalothin and amoxicillin. The *bla*OXA58 gene was first identified in the bacterium *Acinetobacter baumannii*, while *bla*OXA48 was detected in *Klebsiella pneumoniae*. This enzyme and its variants are also widespread in other *Enterobacteriaceae* [49]. Products of the *bla*OXA48 gene are currently the most commonly-detected carbapenemases in Europe, especially in Mediterranean countries [50].

The present study showed that the *bla*TEM1 gene was the most common at all stages of the wastewater-treatment process, which, as mentioned above, is characteristic of clinical sources of contamination, among others [42]. The analysis of the occurrence of *bla*TEM1 genes in bacteria isolated at different stages of wastewater treatment showed that: in raw wastewater they were present in 45% of strains; in the aeration chambers their frequency was almost 84%; and in treated wastewater their frequency was 94% (Figure 4).

The primers used in the study for the PCR reaction were synthesized based on the sequences dedicated to the corresponding antibiotic-resistance determinant genes. The next stage of the study, concerning the sequencing of the relevant DNA fragments, showed that the sequences of the PCR products for the *bla*GES and the *bla*PER genes were not identical to the reference sequences of these genes. This could be due to the genetic variability within the genes present in the strains isolated from the wastewater-treatment plant, or could suggest an imperfection in the primers dedicated to the *bla*PER and the *bla*GES genes; however, this would require further detailed studies. In the latter case, one would indeed have to reckon with the fact that the results obtained for these genes might not be entirely reliable. Moreover, it suggests that sequencing of the PCR reaction products may be essential to obtain reliable identification of individual resistance genes and to avoid false-positive results.

The number of copies of *bla*TEM1 gene present in wastewater determined by qPCR reached 10^5^–10^6^/cm^3^, which was higher than the 1.26–10^4^ copies of the *bla*TEM gene observed by Hembach et al. [44] in treated wastewater samples from a German wastewater-treatment plant. Its widespread occurrence in urban wastewater, especially after the treatment process, could pose a significant health risk. In addition, nearly 37% of isolates from treated wastewater contained the *bla*OXA58 gene, which encodes a class D carbapenemase found in clinical isolates of *Pseudomonas mirabilis*, among others [51]. Bacterial resistance to carbapenem antibiotics, is a particularly dangerous phenomena, as they are often considered “last resort” drugs used to treat severe bacterial infections [52,53].

The study also considered the presence of class I (*Int*1) and class III (*Int* 3) integrons. As suggested by Tacao et al. [54], class I integrons are more easily exchanged and disseminated among strains characterized by the activity of extended-spectrum β-lactamases. At the same time, they are more frequently detected in environmental samples compared with class II and III integrons [55]. Although integrons are not considered mobile genetic elements per se, their localization on plasmids and transposons gives them the ability to transmit multi-resistance traits [56]. In the present study, we observed both the abundant presence of the *Int*1 gene (35.5% of isolates from the aeration chambers) and the co-occurrence of the *bla*TEM1 gene and class I integron. A similar relationship was observed during their study by Li et al. [57].

Mobile genetic elements—plasmids, transposons and integrons—are important factors when it comes to horizontal gene transfer, among other things, contributing to the phenomena of multi-resistance formation. The integrons responsible for the capture and the expression of antibiotic-resistance genes are often detected in wastewater-treatment plants. The most common integrons in wastewater-treatment plants are class 1 integrons [58], representatives of which were also isolated in the present study). Various types of plasmids commonly found in the biocenosis of bacteria that make up the microflora in wastewater-treatment plants play an important role in the transfer of antibiotic-resistance genes. Li et al. [59] conducted a study, in which they showed that plasmid metagenomes obtained at a wastewater-treatment plant contained numerous antibiotic-resistance genes, confirming that the wastewater-treatment plant can be a site for the spread of drug-resistance genes located on plasmids. Zhang et al. [58] used the transposon-aided capture system (TRACA) to isolate novel plasmids responsible for antibiotic resistance in bacteria from activated sludge. Li et al. [59] have isolated as many as 16 new plasmids determining bacterial resistance in a wastewater-treatment plant.

It should be also considered that bacterial resistance to antibiotics can be determined not only genetically, thanks to the presence of specific antibiotic-resistance genes. Non-genetic antibiotic resistance can be non-inherited (non-inherited resistance) or it can be completely gene-independent (non-genetic inheritance). Non-genetic inheritance results from the expression of a drug-resistance gene in a cell that has acquired resistance through a process of transformation with foreign DNA containing the drug-resistance gene. Resistance acquired by cells resulting from the division of a transformed parent cell can be both gene-driven (in cells that have inherited the genetic factor), and non-genetic (in cells that have received only gene expression products [60]. Non-inherited resistance, on the other hand, functions through several different mechanisms. One of them is an antibiotic indifference, which occurs in bacteria that do not have drug-resistance genes but are insensitive to antibiotics due to the fact that they are not undergoing cell divisions at any given time, or they are living in an environment that does not provide sufficient nutrients for their metabolism. This is especially true for bacteria in the growth inhibition phase. Another mechanism is known as persistence, or natural insensitivity to antibiotics, which occurs in some bacterial populations. The cells of these bacteria do not contain drug-resistance genes and genetically do not differ from other cells of the same population, showing a sensitivity to a given antibiotic. Again, the cause is believed to be a temporary inhibition of replication and cell division in some representatives of a given bacterial population. Non-inherited resistance can also be associated with the formation of a biofilm by bacteria, which performs a protective function against cells thanks to a polysaccharide matrix and the presence of other cells, limiting their direct exposure to antibiotics and thus decreasing the effectiveness of antibiotics [61].

Available literature data confirm that bacteria present in wastewater exhibit β-lactamase-like enzyme activity [62]. In the present study, an attempt was made to assess the magnitude of the occurrence of these enzymes at different stages of the wastewater-treatment process. Applying the double-disc method, we detected the presence of β-lactamases only in some of the isolates tested. The results obtained from the double-disc tests suggested that bacterial isolates most often exhibited metallo-β-lactamase activity, while carbapenemases, cephalosporinases and extended substrate-spectrum β-lactamases were almost twice as rare, being detected in only about 10% of wastewater samples. The issue of ESBL detection is particularly relevant due to the fact that they are present in many *Enterobacteriaceae* bacteria commonly found in wastewater, and enable hydrolysis of penicillin, ceftazidime, cefotaxime, and monobactams such as aztreonam and fourth-generation cephalosporins, among others [63,64]. The double-disc test showed no carbapenamase or cephalosporinase activity in any of the strains tested from treated wastewater. Most enzymes were detected in wastewater flowing into the treatment plant. Strains characterized by the activity of at least one of the enzymes accounted for as much as 40–50% of the total bacteria present in some raw wastewater streams.

Comparing the above enzymatic results with the results of the analysis of the genetic potential of the bacteria, it can be concluded that the double-disc method (used for clinical purposes, among others) allowed the detection of the presence of β-lactamases only in a part of the tested isolates and is perhaps not sensitive enough, especially in the case of strains isolated from technological systems. The confirmation of this thesis can be found in the fact that the use of the Blue-Carba test, recommended as a test with very high sensitivity (98–100%) [65] and dedicated to the detection of carbapenemases in Gram-negative bacteria, allowed the detection of carbapenemase activity in 30–50% of isolates, but in as many as 97% of strains previously found to be multi-resistant. It is clear from this that the ability to detect the activity of enzymes that determine antibiotic resistance depends largely on the choice of appropriate testing procedures.

As the results of numerous studies show, bacterial resistance to antibiotics does not only affect clinical conditions or environmental elements (water and land), but also technological systems, including wastewater-treatment plants. An anthropogenic pressure promotes the spread of drug-resistant bacteria and genes that determine antibiotic resistance [66]. Antibiotic-resistant bacteria are becoming a quantitatively important component of the biocenoses populating biological wastewater-treatment systems. The research carried out in the present study showed a significant share of β-lactam antibiotic-resistant strains in the microflora of an urban wastewater-treatment plant (reaching several tens of percent), and its increase during the treatment process. It also allowed quantitative analysis of the genetic and enzymatic determinants of antibiotic resistance, confirming the prevalence of this phenomenon. The detected variants of antibiotic-resistance genes showed similarity in samples taken at different stages of the treatment process, confirming the possibility of their spread and passage into the treated wastewater. As the study was conducted over a period of three years, it can be concluded that the observed results are not incidental but they reflect a more permanent phenomena. The fluctuations in the abundance of antibiotic-resistant bacteria and their percentage of the total number of bacteria during the period studied, and the fact that no upward trend was observed over the three years suggest that the main problem seems to be the rise of the multi-resistance trait in the microbial community of bacteria in the wastewater-treatment process.

## 5. Conclusions

The aeration chambers in the wastewater-treatment plant under study were identified as a site for the spread of the bacterial resistance to the selected β-lactam antibiotics, as confirmed by both the quantitative studies of the resistant microflora and the analysis of the occurrence of a number of drug-resistance genes. During the process, both the selection of multi-resistant bacteria and the increase in the number of drug-resistance genes in individual isolates were observed. The *bla*TEM1 gene was the most frequently detected of the genes analyzed, with a high level of similarity in the sequence of this gene in the strains isolated at different stages of the wastewater-treatment process. There was no increase in the number of strains exhibiting β-lactamase enzyme activity during the wastewater-treatment process, although the presence of these enzymes was noted in 97% of multi-resistant bacteria. Antibiotic-resistant bacteria were found to pass into the treated wastewater in significant quantities, e.g., the resistance to penicillin was exhibited by more than 50% of the bacteria in the wastewater-treatment plant effluent. The *bla*TEM1 gene was detected in 94% of bacteria in the wastewater after the treatment process.

## Figures and Tables

**Table 1:** Oligonucleotides used as primers in the PCR and qPCR reactions.

Primer	Type of Application	Sequence of Oligonucleotides (5′ > 3′)
GES	FORWARD	CTGGCAGGGATCGCTCACTC
	REVERSE	GGTTTCCGATCAGCCACCTCTCA
PER	FORWARD	CAGTGTGGGGGCCTGACGAT
	REVERSE	CTGAGCAACCTGCGCAATRATAGCTT
TEM	FORWARD	TCGCCGCATACACTATTCTCAGAATGAC
	REVERSE	CAGCAATAAACCAGCCAGCCGGAAG
OXA 58	FORWARD	GTGCTGAGCATAGTATGAGTCGAGC
	REVERSE	GGTCTACAGCCATTCCCCAGCC
OXA 48	FORWARD	CACCAAGTCTTTAAGTGGGATGGACA
	REVERSE	CCGATACGTGTAACTTATTGTGATACAGCTT
OXA 1	FORWARD	CAACGGATTAACAGAAGCATGGCTCG
	REVERSE	GCTGTRAATCCTGCACCAGTTTTCCC
Int 1	FORWARD	GGTCAAGGATCTGGATTTCG
	REVERSE	ACATGCGTGTAA ATCATCGTC
Int 3	FORWARD	AGTGGGTGGCGAATGAGTG
	REVERSE	TGTTCTTGTATCGGCAGGTG
CTX	FORWARD	ATGTGCAGYACCAGTAARGTKATGGC
	REVERSE	GGTRAARTARGTSACCAGAAYCAGCGG
SHV	FORWARD	TGTATTATCTC(C/T)CTGTTAGCC(A/G)CCCTG
	REVERSE	GCTCTGCTTTGTTATTCGGGCCAAGC
16S	FORWARD	GTGSTGCAYGGYTGTCGTCA
	REVERSE	ACGTCRTCCMCACCTTCCTC

## Data Availability

Not applicable.
